# Parkinsonian Neurotoxins Impair the Pro-inflammatory Response of Glial Cells

**DOI:** 10.3389/fnmol.2018.00479

**Published:** 2019-01-10

**Authors:** Neus Rabaneda-Lombarte, Efren Xicoy-Espaulella, Joan Serratosa, Josep Saura, Carme Solà

**Affiliations:** ^1^Department of Brain Ischemia and Neurodegeneration, Institut d’Investigacions Biomèdiques de Barcelona (IIBB)—Consejo Superior de Investigaciones Científicas (CSIC), Institut d’Investigacions Biomèdiques August-Pi i Sunyer (IDIBAPS), Barcelona, Spain; ^2^Biochemistry and Molecular Biology Unit, School of Medicine, Institut d’Investigacions Biomèdiques August-Pi i Sunyer (IDIBAPS), University of Barcelona, Barcelona, Spain

**Keywords:** glial activation, mixed glia, microglia, immune response, MPP+, rotenone, glial metabolism, Parkinson’s disease

## Abstract

In the case of Parkinson’s disease (PD), epidemiological studies have reported that pesticide exposure is a risk factor for its pathology. It has been suggested that some chemical agents, such as rotenone and paraquat, that inhibit the mitochondrial respiratory chain (in the same way as the PD mimetic toxin 1-methyl-4-phenylpyridinium, MPP+) are involved in the development of PD. However, although the neurotoxic effect of such compounds has been widely reported using *in vivo* and *in vitro* experimental approaches, their direct effect on the glial cells remains poorly characterized. In addition, the extent to which these toxins interfere with the immune response of the glial cells, is also underexplored. We used mouse primary mixed glial and microglial cultures to study the effect of MPP+ and rotenone on glial activation, in the absence and the presence of a pro-inflammatory stimulus (lipopolysaccharide plus interferon-γ, LPS+IFN-γ). We determined the mRNA expression of the effector molecules that participate in the inflammatory response (pro-inflammatory cytokines and enzymes), as well as the nitric oxide (NO) and cytokine production. We also studied the phagocytic activity of the microglial cells. In addition, we evaluated the metabolic changes associated with the observed effects, through the measurement of adenosine triphosphate (ATP) production and the expression of genes involved in the control of metabolic pathways. We observed that exposure of the glial cultures to the neurotoxins, especially rotenone, impaired the pro-inflammatory response induced by LPS/IFN-γ. MPP+ and rotenone also impaired the phagocytic activity of the microglial cells, and this effect was potentiated in the presence of LPS/IFN-γ. The deficit in ATP production that was detected, mainly in MPP+ and rotenone-treated mixed glial cultures, may be responsible for the effects observed. These results show that the response of glial cells to a pro-inflammatory challenge is altered in the presence of toxins inhibiting mitochondrial respiratory chain activity, suggesting that the glial immune response is impaired by such agents. This may have relevant consequences for brain function and the central nervous system’s (CNS’s) response to insults.

## Introduction

Microglia are the main endogenous immune cells of the central nervous system (CNS). Under physiological conditions, they constantly patrol the CNS parenchyma, ready to detect alterations that could interfere with normal brain function. In response to noxious stimuli, microglial cells develop a wide range of reactive phenotypes aimed at re-establishing cerebral homeostasis and minimizing neuronal damage. In this way, they can respond to alterations in the CNS homeostasis due to the presence of exogenous pathogens or anomalous protein aggregates resulting from pathological processes. In addition, they are also able to respond to neuronal damage resulting from brain lesions, brain ischemia, neurodegenerative diseases or exposure to neurotoxic agents (Salter and Stevens, 2017).

In fact, exposure to neurotoxic agents, such as pesticides, may contribute to the development of some neurodegenerative diseases (Mostafalou and Abdollahi, 2013). In 1982, the accidental exposure to 1-methyl-4-phenyl-1,2,3,6-tetrahydropyridine (MPTP) in drug abusers caused parkinsonism (Langston et al., 1983). Epidemiological studies show that exposure to the pesticides rotenone and paraquat, which are functional and structural analogs of MPTP respectively, as well as to other pesticides, is a risk factor for Parkinson’s disease (PD; Tanner et al., 2011; Goldman, 2013; Kamel, 2013). MPTP and its analogs are inhibitors of the mitochondrial respiratory chain and it has been suggested that mitochondrial dysfunction is involved in the induction of oxidative damage in dopaminergic neurons in parkinsonism (Dauer and Przedborski, 2003; Goldman, 2013). Due to the particular sensitivity of dopaminergic neurons to the effect of these neurotoxins, experimental models of PD have been developed by exposing neuronal cell cultures or laboratory animals to these agents (Bové and Perier, 2012). These experimental models are useful for studying mechanisms of dopaminergic neuronal cell degeneration and testing potential therapeutic approaches. However, although the toxic effect of MPTP [or its active metabolite 1-methyl-4-phenylpyridinium (MPP+)] and rotenone on dopaminergic neurons has been widely described using both *in vivo* and *in vitro* approaches, reports of their direct effect on glial cells are scarce. In addition, there is some controversy regarding the results already obtained. Either no direct effect of MPP+ on microglial cell function (Gao et al., 2003; Jin et al., 2012), or an increase in the expression of pro-inflammatory markers in microglial cells after MPP+ exposure (Bournival et al., 2012; Chen et al., 2015) has been reported. Similarly, either an increase in the expression of pro-inflammatory factors (Gao et al., 2013; Yuan et al., 2013; Du et al., 2014) or no direct effect on the production of inflammatory factors (Klintworth et al., 2009) has been observed in rotenone-treated microglial cell cultures.

Since reciprocal communication exists in the CNS between neuronal and glial cells, alterations in neuronal function may affect glial function and vice versa. In fact, a possible role of glial activation in the development of neuronal damage in neurodegenerative diseases has been repeatedly proposed (Perry et al., 2010; Colonna and Butovsky, 2017). In addition, communication also exists between glial cells, and alterations in a given cell type may affect the function of other glial cell types. Consequently, alterations in glial function due to exposure to neurotoxic compounds merit study, especially in the context of neurodegenerative diseases in which such exposure is considered a risk factor. The aim of this study was therefore to characterize the effects of MPP+ and rotenone on glial activation using primary mixed glial cultures, (mainly composed of astrocytes and microglia) and microglial cultures. We determined the direct effect of these neurotoxins on glial cell function, and also whether they could interfere with glial activation induced by a classical pro-inflammatory stimulus such as lipopolysaccharide (LPS)/interferon-γ (IFN-γ). We observed that MPP+ and rotenone did not induce the expression of pro-inflammatory markers by glial cells *per se*. However, the LPS/IFN-γ-induced pro-inflammatory response was modified in glial cultures in the presence of MPP+ and rotenone. These neurotoxins induced modifications in the mRNA expression of pro-inflammatory genes and phagocytic activity. Alterations in adenosine triphosphate (ATP) production could account for the effects observed. These results show that insults affecting the metabolic activity of glial cells, result in an altered immune response, which may have relevant consequences for normal brain function and the CNS response to insults.

## Materials and Methods

Experiments were carried out in accordance with European Union directives (86/609/EU) and Spanish regulations (BOE 67/8509-12, 1988) on the use of laboratory animals, and were approved by the Ethics and Scientific Committees of the University of Barcelona and CSIC.

### Cell Cultures

*Primary mixed glial cultures* were prepared from the cerebral cortex of 1–3-day old C57Bl/6 mice as previously described (Gresa-Arribas et al., 2010). The culture medium used was the Dulbecco’s modified Eagle medium-F12 nutrient mixture (GIBCO) supplemented with 10% heat-inactivated fetal bovine serum (FBS, Invitrogen, Molecular Probes, Eugene, OR, USA), 20 U/mL penicillin-20 μg/mL streptomycin (Invitrogen), and 0.5 μg/mL amphotericin B (Fungizone^®^, Invitrogen). The cells were seeded at a density of 3.5 × 10^5^ cells/mL (100 μL, 300 μL and 2.5 mL per well into 96-, 48- and 6-well culture plates) and cultured at 37°C in a humidified 5% CO_2_ atmosphere. The medium was replaced once a week. The cultures were used at 21 DIV.

*Primary microglia enriched cultures* were obtained from 21 DIV mixed glial cultures using the mild trypsinization method as previously described (Saura et al., 2003). Microglia enriched cultures were used 24 h after isolation by this procedure.

### Cell Culture Treatments

*LPS and IFN-*γ *treatment:* Cells were treated with 100 ng/ml LPS (*E. coli* 026:B6, Sigma-Aldrich, St. Louis, MO, USA) and 0.1 ng/ml IFN-γ (Sigma-Aldrich) for 6 h or 24 h. Stock solutions of 1 mg/mL LPS in a serum-free culture medium and 10 μg/mL IFN-γ in a serum-containing culture medium, were prepared and stored at −20°C.

*MPP+ and rotenone treatment:* Cells were treated with 10, 25, 50 and 100 μM MPP+ or 20, 40, 100 and 150 nM rotenone (both from Sigma-Aldrich) for 6 h or 24 h, in the absence or presence of LPS/IFN-γ. Stock solutions of 50 mM MPP+ in milliQ H_2_O and 10 mM rotenone in DMSO were freshly prepared on the day of treatment. DMSO in the cell cultures was always below 1/1,000.

Treatments were added directly to the culture medium.

### Nitric Oxide Production

Nitric oxide (NO) production was estimated from the nitrite accumulation in the culture supernatant using the colorimetric Griess reaction. Briefly, the culture supernatant from the glial cells seeded into 96-well culture plates, was collected 24 and 48 h after treatments and stored at −20°C until used. Fifty microliter aliquots of the culture supernatant were incubated with equal volumes of the Griess reagent for 10 min at 20–25°C. Optical density at 540 nm was measured using a microplate reader (Multiskan Spectrum, Thermo Fisher Scientific, Vantaa, Finland). Nitrite concentration was determined from a sodium nitrate standard curve.

### Cell Viability Measurements

Glial cells seeded into 96-well culture plates were used to estimate cell viability from the metabolic activity by a 3-(4,5-dimethylthiazol-2-yl)-2,5-diphenyl tetrazolium bromide (MTT) colorimetric assay, 24 h after treatments. Briefly, MTT (Sigma-Aldrich) was added to the cell cultures to reach a final concentration of 1 mg/mL. After incubation for 30 min (mixed glial cultures) or 90 min (microglial cultures) at 37°C, the medium was removed and 200 μL of DMSO was added to each well. The optical density of the resulting blue formazan was measured at 570 nm using a microplate reader (Multiskan Spectrum, Thermo Fisher Scientific). Readings were taken at 650 nm to obtain background levels. Results were expressed as percentages of the control.

Propidium iodide (PI) and Hoechst labeling were performed to corroborate data obtained in the MTT assay. Briefly, cells were incubated with PI (7.5 μg/ml, Molecular Probes, Eugene, OR, USA) and Hoechst 33342 (3 μg/ml, Molecular Probes) for 10 min. Microscopy images were obtained using an Olympus IX70 microscope (Olympus, Okoya, Japan) and a digital camera (CC-12, Olympus Soft Imaging Solutions GmbH, Hamburg, Germany). The extent of cell death was calculated from the ratio between PI positive nuclei, corresponding to dead cells, vs. Hoechst positive total nuclei.

### RNA Extraction and Quantitative Real Time PCR

Glial cells seeded into six-well culture plates were used (one or two wells per experimental condition for mixed glia and microglia, respectively) to assess the mRNA expression of pro-inflammatory markers by quantitative real time polymerase chain reaction (PCR) 6 h after treatments. A High Pure RNA Isolation Kit (Roche Diagnostics Scheiwz AG, Rotkreuz, Switzerland) was used to isolate the total RNA from the mixed glial cultures. A PureLink RNA micro kit (Invitrogen) was used to isolate the total RNA from the primary microglial cultures. The RNA (0.5–1 μg) was reverse transcribed with random primers using the Transcriptor Reverse Transcriptase Kit (Roche Diagnostics). Three nanograms of cDNA were used to perform quantitative real time PCR (qRT-PCR) with the IQ SYBRGREEN SuperMix (Bio-Rad Laboratories, Hercules, CA, USA) using an iCycler MyIQ apparatus (Bio-Rad Laboratories) as previously described (Dentesano et al., 2014). The primers used (Integrated DNA Technology, IDT, Skokie, IL, USA) are shown in Table 1. Samples were run for 40 cycles (95°C for 15 s, 60°C for 30 s, and 72°C for 15 s). The amplification specificity was confirmed by the analysis of melting curves. Relative gene expression values were calculated using the ΔΔCt method (Livak and Schmittgen, 2001). β-Actin and 18S ribosomal RNA (Rn18s) were used as the reference genes.

**Table 1:** Primers used for quantitative real time polymerase chain reaction (qRT-PCR).

Target mRNA	Accession number	Forward primer (5′→3′)	Reverse primer (5′→3′)
Carkl	NM_029031.3	CAGGCCAAGGCTGTGAAT	GCCAGCTGCATCATAGGACT
COX2	NM_011198.4	TGCAGAATTGAAAGCCCTCT	CCCCAAAGATAGCATCTGGA
Glut1	NM_011400.3	CATCCTTATTGCCCAGGTGTTT	GAAGATGACACTGAGCAGCAGA
gp91phox	NM_007807.5	ACTCCTTGGGTCAGCACTGGCT	GCAACACGCACTGGAACCCCT
Hifα	NM_010431.2	ACAAGTCACCACAGGACAG	AGGGAGAAAATCAAGTCG
Hk1	NM_010438.3	GATGGAGGTGAAGAAGAAGC	GGAAACGAGAAGGTGAAGC
Hk2	NM_013820.3	CGGTACACTCAATGACATCC	GTAGACAGAGCCATCCACG
IL1β	NM_008361.4	TGGTGTGTGACGTTCCCATTA	CAGCACGAGGCTTTTTTGTTG
IL6	NM_031168.2	CCAGTTTGGTAGCATCCATC	CCGGAGAGGAGACTTCACAG
iNOS	NM_010927.3	GGCAGCCTGTGAGACCTTTG	GCATTGGAAGTGAAGCGTTTC
Pfkp	NM_019703.4	AAGCTATCGGTGTCCTGACC	TCCCACCCACTTGCAGAAT
TNFα	NM_013693.3	TGATCCGCGACGTGGAA	ACCGCCTGGAGTTCTGGAA
*Reference genes*:
β-Actin	NM_007393.5	CAACGAGCGGTTCCGATG	GCCACAGGATTCCATACCCA
Rn18s	NR_003278.3_	GTAACCCGTTGAACCCCATT	CCATCCAATCGGTAGTAGCG

*β-Actin, Actin, beta; Carkl, sedoheptulokinase; COX2, cyclooxygenase-2; Glut1, Glucose transporter 1; gp91phox, catalytic subunit of NAPH oxidase; Hifα, hypoxia inducible factor 1, alpha subunit; Hk1, hexokinase 1, Hk2, hexokinase 2; IL1β, interleukin 1, beta; IL6, interleukin 6; iNOS, inducible nitric oxide synthase; Pfkp, phosphofructokinase, platelet; TNFα, tumor necrosis factor alpha; Rn18s, 18S ribosomal RNA*.

### ELISAs

The interleukin (IL) 1β, IL6 and tumor necrosis factor α (TNFα) release in the culture supernatant was determined using ELISA kits specific for each cytokine (mouse IL1-β ELISA Ready-SET-GO!, mouse IL6 ELISA Ready-SET-GO! and mouse TNFα ELISA Ready-SET-GO!, eBioscience-Affimetrix, Inc., San Diego, CA, USA), following the manufacturer’s instructions. The culture supernatant from 48-well culture plates was collected 24 h after treatments and stored at −80°C until use. IL1β, IL6 and TNFα concentrations were determined from the standard curves.

### Phagocytosis Assay

The phagocytic activity of the microglial cells was assessed 24 h after treatments. Briefly, the microglial cell cultures in 48-well plates were incubated for 1 h at 37°C with fluorescent latex beads (FluoSpheres, carboxylate-modified microspheres, 2.0 μm, red fluorescent (580/605), 2% solids; Thermofisher Scientific; 1/1,000) 23 h after treatments. Then, the cells were washed three times with a phosphate-buffered saline (PBS) and fixed with 4% paraformaldehyde for 15 min.

Immunocytochemistry was performed using a rabbit polyclonal anti-Iba1 primary antibody (1/500, WAKO; Japan), a specific marker for microglial cells. Cells were first incubated with 0.3% Triton-X-100 in PBS containing 1% bovine serum albumin (BSA) and 10% normal donkey serum for 20 min at room temperature, and then overnight at 4°C with the primary antibody. Once they had been rinsed in PBS, cells were incubated for 1 h at room temperature with an ALEXA 488 donkey anti-rabbit secondary antibody (1/1,000; Invitrogen). Antibodies were diluted in 0.3% Triton X-100 in PBS containing 1% BSA and 10% normal donkey serum.

Images of three microscopic fields using a 20× objective were obtained with an Olympus IX70 fluorescence microscope and a digital camera (CC-12, Olympus Soft Imaging Solutions GmbH). Two to three wells per experimental condition were processed and each experimental condition was repeated at least four times. Visual counting of the FluoSpheres was performed. The percentage of phagocytic cells and the average number of fluorescent microspheres per microglial cell were calculated. To further characterize the phagocytic activity, we also calculated the percentage of cells showing lower phagocytic activity (microspheres/cell) than the controls and the percentage of cells showing higher phagocytic activity than the controls.

### ATP Production

The intracellular production of ATP was determined using a luminescence assay kit (ATPlite Luminescence ATP Detection Assay System, PerkinElmer, Waltham, MA, USA) following the manufacturer’s instructions. Briefly, cells in 96-well plates (mixed glia) or 6-well-plates (microglia) were lysed 24 h after treatments and the ATP concentration was measured based on the production of light, caused by the reaction of the ATP with added luciferase and D-luciferin. The emitted light was quantified using a luminometer (Orion Microplate Luminometer, Berthold Detection System, Germany). The ATP concentration in the samples was calculated from an ATP standard curve.

### Data Presentation and Statistical Analysis

The results are presented as the mean + SEM. At least three independent experiments were performed for analysis. Data were statistically analyzed with the GraphPad Prism software. Statistical analyses were performed using the one-way analysis of variance (ANOVA) followed by the Newman-Keuls post-test, and a two-way ANOVA followed by the Bonferroni post-test. Values of *p* < 0.05 were considered statistically significant.

## Results

### Effects of MPP+ and Rotenone on Glial Cell Viability

**Figure 1:**
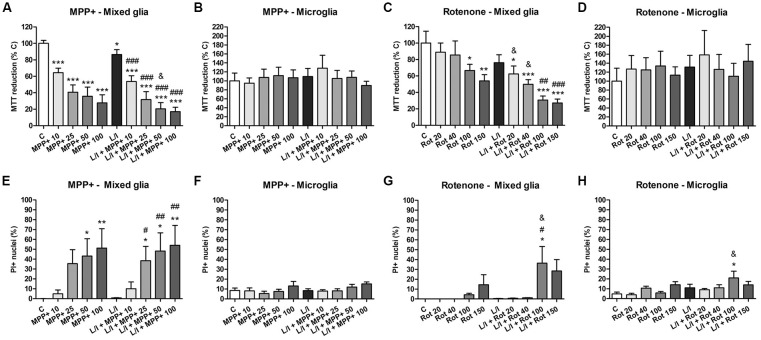
Effect of 1-methyl-4-phenylpyridinium (MPP+) and rotenone on glial cell viability. **(A–D)** MPP+ and rotenone induced alterations in 3-(4,5-dimethylthiazol-2-yl)-2,5-diphenyltetrazolium bromide (MTT) reduction in the primary glial cultures. Effect of 10, 25, 50 and 100 μM MPP+ treatment for 24 h on the mixed glial cultures **(A)** and microglial cultures **(B)**, both in the absence and the presence of lipopolysaccharide (LPS)/interferon-γ (IFN-γ; L/I). Effect of 20, 40, 100 and 150 nM rotenone (Rot) treatment for 24 h on the mixed glial cultures **(C)** and microglial cultures **(D)**, both in the absence and the presence of L/I. **(E–H)** Percentage of propidium iodide (PI) positive nuclei in the mixed glial cultures **(E)** and microglial cultures **(F)** treated for 24 h with 10, 25, 50 and 100 μM MPP+, both in the absence and the presence of LPS/IFN-γ (L/I). Percentage of PI positive nuclei in the mixed glial cultures **(G)** and microglial cultures **(H)** treated for 24 h with 20, 40, 100 and 150 nM rotenone (Rot), both in the absence and the presence of L/I. Bars are means ± SEM of four independent experiments. *p < 0.05, **p < 0.01 and ***p < 0.001 vs. control (C); ^#^*p* < 0.05, ^##^*p* < 0.01 and ^###^*p* < 0.001 vs. L/I; ^&^*p* < 0.05 vs. MPP+ or Rot alone; one-way analysis of variance (ANOVA; repeated measures) and Newman-Keuls post-test.

In a preliminary study, we performed dose-response experiments in order to select working concentrations of MPP+ and rotenone that did not result in significant alterations in cell viability after 24 h exposure. We evaluated glial cell viability after treating the mixed glial or the microglial cultures with increasing concentrations of MPP+ (10, 25, 50 and 100 μM) or rotenone (20, 40, 100 and 150 nM), both in the absence and in the presence of LPS/IFN-γ, considering the MTT assay and PI staining. In mixed glial cell cultures, MPP+ induced a concentration-dependent decrease in MTT reduction that was accentuated in the presence of LPS/IFN-γ (Figure 1A). On the contrary, no alterations in MTT reduction were observed in microglial cell cultures treated with MPP+, both in the absence and presence of LPS/IFN-γ (Figure 1B). Rotenone-treated mixed glial cell cultures showed a significant decrease in MTT reduction from 100 nM rotenone. In the presence of LPS/IFN-γ, there was a significant decrease in MTT reduction even at 20 nM rotenone (Figure 1C). As in the case of MPP+ treatments, no alterations in MTT reduction were observed in microglial cell cultures treated with rotenone or rotenone and LPS/IFN-γ (Figure 1D). To determine whether the decrease in MTT reduction in MPP+ and rotenone-treated mixed glial cell cultures was due to a decrease in metabolic activity or due to cell death, PI staining was performed. Mixed glial cultures treated with 50 and 100 μM MPP+ showed a significant increase in the percentage of PI positive nuclei. This effect was accentuated in the presence of LPS/IFN-γ, and a significant increase was also detected at 25 μM MPP+ (Figure 1E). No alterations in the percentage of PI-positive nuclei were observed in microglial cultures treated with MPP+ (Figure 1F). In addition, no significant increases in the percentage of PI-positive nuclei were observed in rotenone-treated mixed glial cell cultures or microglial cultures (Figures 1G,H), with the exception of cells treated with 100 μM rotenone and LPS/IFN-γ.

**Figure 2:**
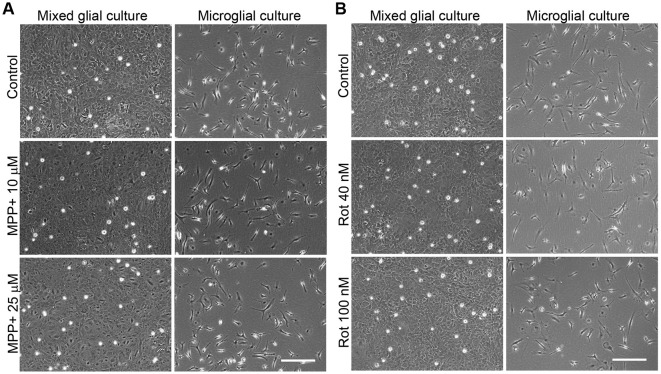
Phase contrast images of the MPP+- and rotenone-treated primary glial cultures. Images show the appearance of the mixed glial cultures and microglial cultures treated for 24 h with 10 and 25 μM MPP+ **(A)** or 40 and 100 nM rotenone (Rot; **B**), the working concentrations used in further studies. Bar = 200 μm.

The concentrations of 10 and 25 μM MPP+ and 40 and 100 nM rotenone were used in subsequent studies. Representative images of the cultures in these experimental conditions are shown in Figure 2, which corroborate the lack of a toxic effect of the concentrations of MPP+ (Figure 2A) and rotenone (Figure 2B) selected for further experiments.

### MPP+ and Rotenone Induce Alterations in the Expression of Pro-inflammatory Genes in LPS/IFN-γ-Treated Primary Glial Cultures

**Figure 3:**
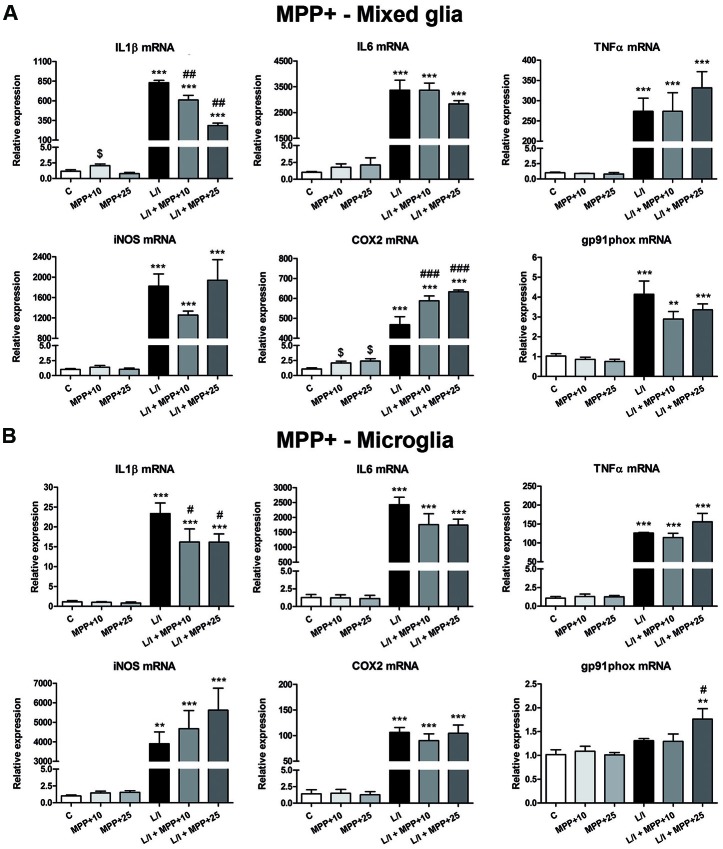
Effect of MPP+ treatment on the mRNA expression of pro-inflammatory markers. mRNA expression of pro-inflammatory cytokines [interleukin-1β (IL-1β), IL-6 and tumor necrosis factor-α (TNF-α)] and enzymes [inducible nitric oxide synthase (iNOS), cyclooxygenase-2 (COX-2), gp91phox] in the primary mixed glial cultures **(A)** and microglial cultures **(B)** treated for 6 h with 10 and 25 μM MPP+, both in the absence and the presence of LPS/IFN-γ (L/I). 18S ribosomal RNA (Rn18s) and β-actin were used as housekeeping genes. Bars are means ± SEM of four independent experiments. **p < 0.01 and ***p < 0.001 vs. C; ^#^*p* < 0.05, ^##^*p* < 0.01 and ^###^*p* < 0.001 vs. L/I; one-way ANOVA and Newman-Keuls post-test. ^$^*p* < 0.05 MPP+ alone vs. C, one-way ANOVA and Newman-Keuls post-test only considering the L/I-free groups. This latter analysis was performed to detect whether the high values observed in the L/I group may hinder the detection of statistical significance of the effects of MPP+ alone.

**Figure 4:**
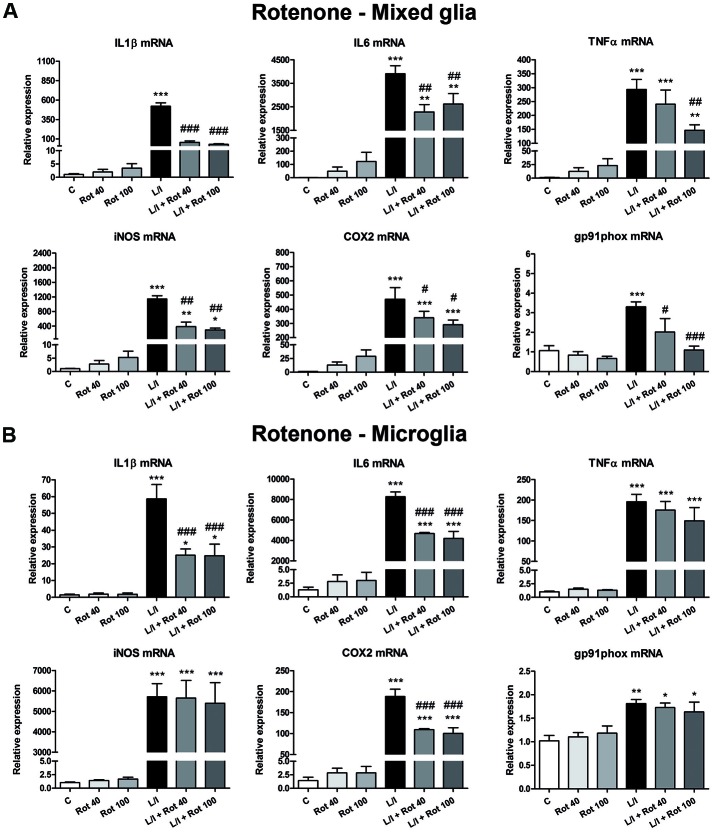
Effect of rotenone treatment on the mRNA expression of pro-inflammatory markers. mRNA expression of pro-inflammatory cytokines (IL-1β, IL-6 and TNF-α) and enzymes (iNOS, COX-2, gp91phox) in the primary mixed glial cultures **(A)** and microglial cultures **(B)** treated for 6 h with 40 and 100 nM rotenone (Rot), both in the absence and the presence of LPS/IFN-γ (L/I). Rn18s and β-actin were used as housekeeping genes. Bars are means ± SEM of four independent experiments. *p < 0.5, **p < 0.01 and ***p < 0.001 vs. control (C); ^#^*p* < 0.05, ^##^*p* < 0.01 and ^###^*p* < 0.001 vs. L/I; one-way ANOVA and Newman-Keuls post-test.

We next determined whether MPP+ and rotenone induced a pro-inflammatory phenotype in primary glial cell cultures, as well as whether they had some effect on the development of the pro-inflammatory response induced by LPS/IFN-γ. We determined the mRNA expression of the cytokines IL1β, IL6 and TNFα and the enzymes inducible NO synthase (iNOS), cyclooxygenase 2 (COX2) and gp91phox (the catalytic subunit of NADPH oxidase), as markers of a pro-inflammatory response. In general, MPP+ (Figure 3) and rotenone (Figure 4) treatment did not significantly induce the mRNA expression of these pro-inflammatory markers in the primary glial cell cultures, although a trend towards increased expression was observed for some mRNAs, especially in rotenone-treated mixed glial cultures. On the contrary, 6 h after LPS/IFN-γ treatment, the mRNA expression of all the pro-inflammatory markers tested was clearly induced (Figures 3, 4). However, MPP+ and especially rotenone induced alterations in the pattern of expression of these markers in LPS/IFN-γ treated cultures. When glial cell cultures were treated with LPS/IFN-γ in the presence of MPP+, the induction of IL1β mRNA expression was significantly inhibited in the mixed glial and microglial cultures (Figures 3A,B), while COX2 mRNA expression was further increased in the mixed glial cultures (Figure 3A) and gp91phox mRNA was induced in the microglial cultures (Figure 3B). More importantly, rotenone exposure significantly abrogated LPS/IFN-γ induction of the mRNA expression of all pro-inflammatory markers in the mixed glial cultures (Figure 4A), as well as IL1β, IL6 and COX2 mRNA expression in microglial cultures (Figure 4B).

### MPP+ and Rotenone Inhibit LPS/IFN-γ- Induced NO and Pro-inflammatory Cytokine Production in Primary Glial Cultures

**Figure 5:**
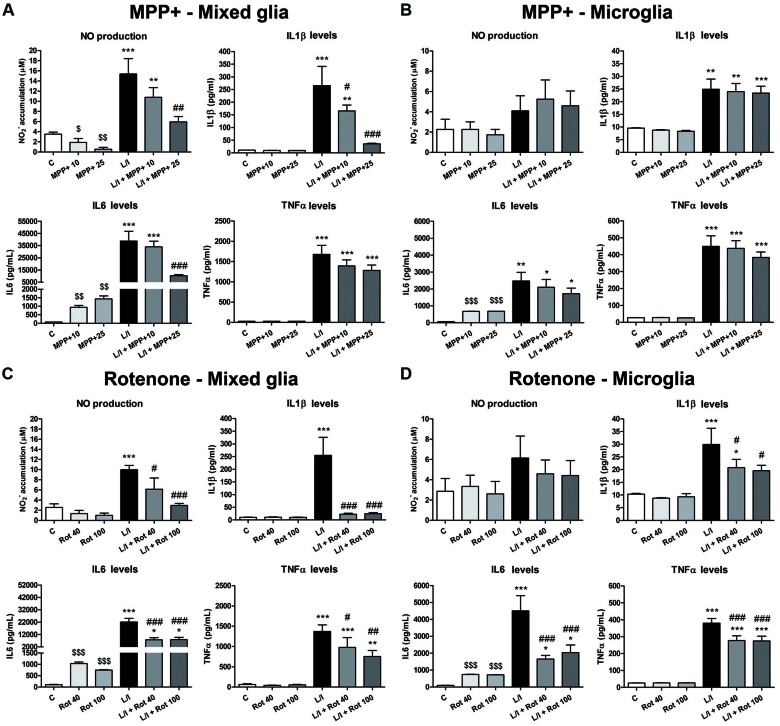
Nitric oxide (NO) and pro-inflammatory cytokine production in the primary glial cell cultures treated with MPP+ and rotenone. NO production was estimated from nitrite accumulation and IL1β, IL6 and TNFα levels were determined by ELISA in the culture medium of the MPP+-treated mixed glial **(A)** and microglial **(B)** cultures, and the rotenone (Rot)-treated mixed glial **(C)** and microglial **(D)** cultures. The cell cultures were treated with 10 and 25 μM MPP+ or 40 and 100 nM Rot for 24 h, in the absence or in the presence of LPS/IFN-γ (L/I). Bars are means ± SEM of four independent experiments. *p < 0.5, **p < 0.01 and ***p < 0.001 vs. control (C); ^#^*p* < 0.05, ^##^*p* < 0.01 and ^###^*p* < 0.001 vs. L/I; one-way ANOVA and Newman-Keuls post-test. ^$^*p* < 0.05, ^$$^*p* < 0.01 and ^$$$^*p* < 0.001 MPP+ and Rot alone vs. C, one-way ANOVA and Newman-Keuls post-test only considering the L/I-free groups. This latter analysis was performed to detect whether the high values observed in the L/I group may hinder the detection of a statistical significance of the effects of MPP+ and Rot alone.

We also analyzed the effect of MPP+ and rotenone on NO, IL1β, IL6 and TNFα release into the culture medium. MPP+ alone induced a decrease in NO production and an increase in IL6 release into the culture medium in mixed glial cell cultures 24 h after treatment (Figure 5A). The latter effect was also observed in the MPP+-treated microglial cultures (Figure 5B), as well as in the rotenone-treated mixed glial (Figure 5C) and microglial cultures (Figure 5D). LPS/IFN-γ-treatment clearly increased NO production in the mixed glial cultures, and MPP+ (25 μM) and rotenone (40 and 100 nM) significantly inhibited this effect (Figures 5A,C). Significant NO production was not detected in the microglial cultures treated with LPS/IFN-γ for 24 h (Figures 5B,D). However, an increase in NO production was observed when the microglial cultures were treated with LPS/IFN-γ for 48 h, but MPP+ and rotenone did not modify this effect (data not shown). With regards to cytokine release, LPS/IFN-γ-treatment resulted in drastic increases in IL1β, IL6 and TNFα levels in the mixed glial (Figures 5A,C) and microglial (Figures 5B,D) cultures. MPP+ exposure (25 μM) significantly inhibited LPS/IFN-γ-induced release of IL1β and IL6, but not TNFα, in the mixed glia (Figure 5A), while it had no significant effect on the production of these cytokines in the microglial cell cultures (Figure 5B). Interestingly, rotenone (40 and 100 nM) significantly inhibited IL1β, IL6 and TNFα release induced by LPS/IFN-γ in both the mixed glial (Figure 5C) and microglial cultures (Figure 5D).

### MPP+ and Rotenone Treatment Inhibit the Phagocytic Activity of Microglial Cells

**Figure 6:**
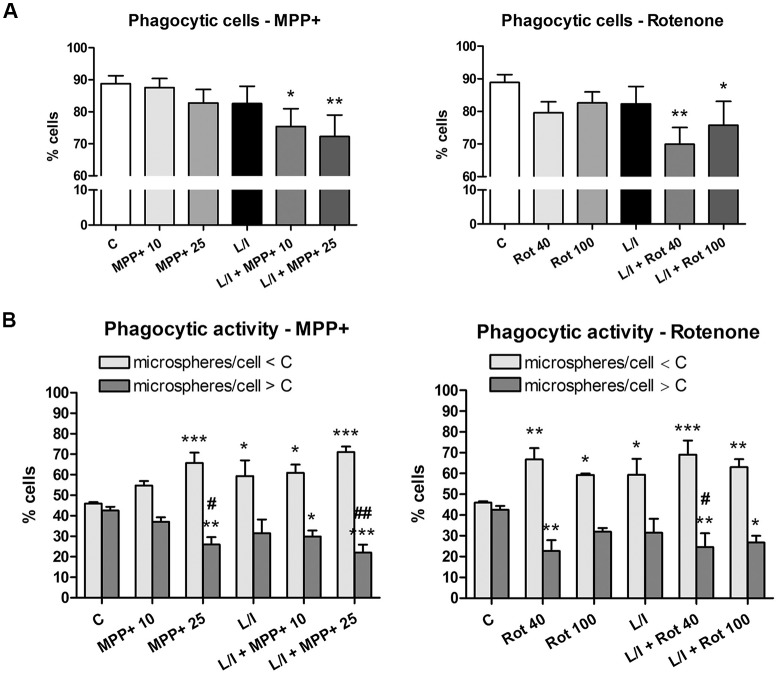
Effect of MPP+ and rotenone treatment on the microglial cell phagocytosis in the primary microglial cell cultures. Phagocytic activity was evaluated through the ingestion of fluorescent microspheres after treating the cell cultures with 10 and 25 μM MPP+ or 40 and 100 nM rotenone (Rot) for 24 h, in both the absence and presence of LPS/IFN-γ (L/I). Internalization of microspheres was quantified after immunofluorescence labeling of the microglial cells using an anti-Iba1 antibody. **(A)** Percentage of cells with microspheres. Bars are means ± SEM of four independent experiments. *p < 0.05 and **p < 0.01 vs. control (C); one-way ANOVA and Newman-Keuls post-test. **(B)** Percentage of cells with low phagocytic activity (number of microspheres/cell < C) and cells with high phagocytic activity (number of microspheres/cells > C). Bars are means ± SEM of four independent experiments. *p < 0.05, **p < 0.01 and ***p < 0.001 vs. corresponding C; ^#^*p* < 0.05 and ^##^*p* < 0.01 vs. low-phagocytic cells; two-way ANOVA and Bonferroni post-test.

We then evaluated whether MPP+ and rotenone modified the phagocytic activity of the microglial cells, another important parameter used to characterize the microglial activation phenotype. Both MPP+ and rotenone treatment showed a tendency to decrease the percentage of phagocytic microglial cells that were statistically significant when the cells were also treated with LPS/IFN-γ (Figure 6A). In addition, MPP+ and rotenone treatment resulted in a significant increase in the percentage of microglial cells showing low phagocytic activity (number of microspheres per cell lower than control) and a subsequent significant decrease in the percentage of cells showing high phagocytic activity (number of microspheres per cell higher than control; Figure 6B). These effects were accentuated in the presence of LPS/IFN-γ.

### ATP Production Is Compromised in MPP+- and Rotenone-Treated Glial Cell Cultures

**Figure 7:**
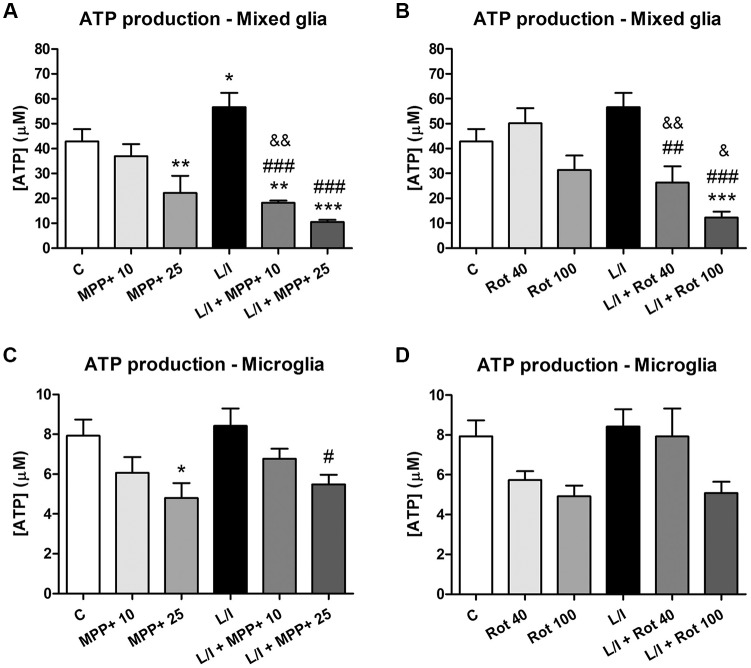
Adenosine triphosphate (ATP) production in the primary glial cell cultures treated with MPP+ and rotenone. Intracellular ATP production was determined in the mixed glial cultures and microglial cultures treated with 10 and 25 μM MPP+ **(A,B)** or 40 and 100 nM rotenone (Rot; **C,D**) for 24 h, in the absence and in the presence of LPS/IFN-γ (L/I). Bars are means ± SEM of five independent experiments. *p < 0.5, **p < 0.01 and ***p < 0.001 vs. control **(C)**; ^#^*p* < 0.05, ^##^*p* < 0.01 and ^###^*p* < 0.001 vs. L/I; ^&^*p* < 0.05 and ^&&^*p* < 0.01 vs. MPP+ or Rot alone; one-way ANOVA and Newman-Keuls post-test.

In an attempt to better characterize the metabolic status of the cells, we determined the intracellular ATP production in response to MPP+, rotenone and LPS/IFN-γ treatments. In general, ATP production was modified in the mixed glial cultures in our MPP+ and rotenone experimental models (*p* < 0.001, one-way ANOVA; Figure 7). In particular, ATP production was significantly decreased after 25 μM MPP+ treatment (Figure 7A). On the contrary, a significant increase in ATP production was detected in the LPS/IFN-γ-treated mixed glial cultures, which was abrogated in the presence of MPP+ and rotenone (Figures 7A,B). ATP production was also modified in the microglial cultures in the MPP+ and rotenone experimental models (*p* < 0.05, one-way ANOVA), but to a lesser extent than in the mixed glial cultures (Figures 7C,D).

### Metabolic Changes in LPS/IFN-γ-Treated Glial Cultures: Effect of MPP+ and Rotenone

**Figure 8:**
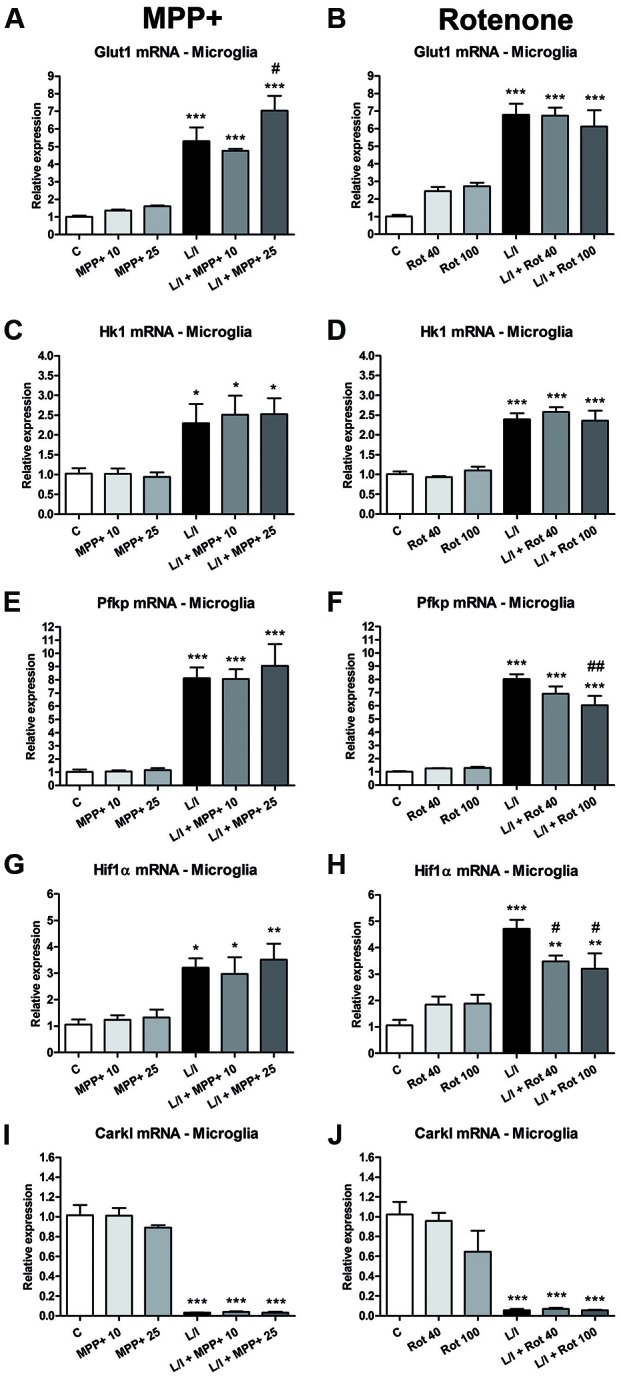
Effect of MPP+ and rotenone treatment on the expression of genes involved in the control of the glycolysis in the microglial cell cultures. mRNA expression of the glucose transporter (Glut1; **A,B**) and the glycolytic enzymes hexokinase 1 (Hk1; **C,D**) and phosphofructokinase 1 (Pfk1; **E,F**), as well as Hif1α **(G,H)** and carbohydrate kinase-like protein 1 (Carkl; **I,J**). The primary microglial cultures were treated for 6 h with 10 and 25 μM MPP+ or 40 and 100 nM rotenone (Rot), both in the absence and the presence of LPS/IFN-γ (L/I). Rn18s and β-actin were used as housekeeping genes. Bars are means ± SEM of four independent experiments. *p < 0.05, **p < 0.01 and ***p < 0.001 vs. control (C); ^#^*p* < 0.05 and ^##^*p* < 0.01 vs. L/I; one-way ANOVA and Newman-Keuls post-test.

**Figure 9:**
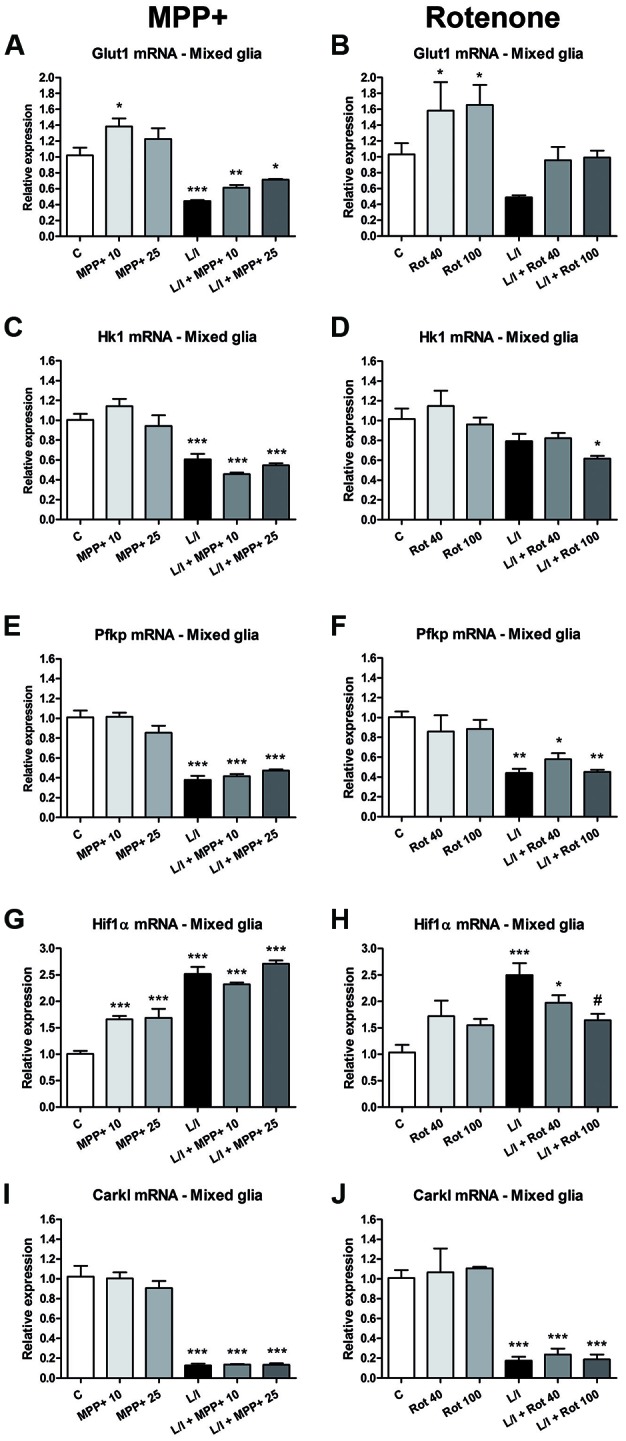
Effect of MPP+ and rotenone treatment on the expression of genes involved in the control of glycolysis in the mixed glial cell cultures. mRNA expression of the glucose transporter Glut1 **(A,B)** and the glycolytic enzymes Hk1 **(C,D)** and Pfk1 **(E,F)**, as well as Hif1α **(G,H)** and Carkl **(I,J)**. The primary mixed glial cultures were treated for 6 h with 10 and 25 μM MPP+ or 40 and 100 nM rotenone (Rot), both in the absence and the presence of LPS/IFN-γ (L/I). Rn18s and β-actin were used as housekeeping genes. Bars are means ± SEM of four independent experiments. *p < 0.05, **p < 0.01 and ***p < 0.001 vs. control (C); ^#^*p* < 0.05 vs. L/I; one-way ANOVA and Newman-Keuls post-test.

In immune cells, the development of specific immune responses is associated with specific metabolic changes. Increased glycolysis and potentiation of the pentose phosphate pathway, together with the inhibition of oxidative phosphorylation has been reported for immune cells showing a pro-inflammatory phenotype. We checked whether this was the case in our glial cultures treated with LPS/IFN-γ and whether MPP+ and rotenone were able to modify it. We determined the mRNA expression of genes encoding critical proteins for the switch to glycolysis: glucose transporter 1 (Glut1; glucose entrance into the cell), key glycolytic enzymes such as hexokinase 1 (Hk1) (glycolysis initial rate limiting step) and phosphofructokinase 1 (PFK1) (master regulator of glycolysis), the glycolysis activator hypoxia-inducible factor 1α (Hif1α) and carbohydrate kinase-like protein (Carkl), involved in the control of the pentose phosphate pathway. In the microglial cell cultures, MPP+ and rotenone alone did not modify the expression of these genes *per se* (Figure 8). On the contrary, as expected, LPS/IFN-γ treatment induced an increase in their expression (Figure 8), with the exception of Carkl mRNA (Figures 8I,J), which showed a decrease. MPP+ treatment further increased LPS-IFN-γ-induced Glut1 mRNA expression (Figure 8A), while rotenone inhibited LPS-IFN-γ-induced Pfk1 (Figure 8F) and Hif1α (Figure 8H) mRNA expression. We also evaluated the expression of these mRNAs in the mixed glial cultures. MPP+ and rotenone alone increased the Glut1 mRNA (Figures 9A,B), MPP+ and Hif1α mRNA expression (Figure 9G). LPS/IFN-γ treatment inhibited the expression of the glycolytic genes Glut1 (Figures 9A,B), Hk1 (Figures 9C,D) and Pfk1 (Figures 9E,F), as well as Carkl mRNA expression (Figures 9I,J), and increased the expression of Hif1α (Figures 9G,H). Rotenone inhibited LPS/IFN-γ-induced Hif1α mRNA expression (Figure 9H).

## Discussion

In this study, we show that the response of glial cells to a pro-inflammatory stimulus is modified by the neurotoxic agents MPP+ and rotenone. MPP+ and rotenone treatment did not induce a significant pro-inflammatory phenotype in the primary mixed glial and microglial cultures *per se*. However, these neurotoxic agents, mainly rotenone, did impair the development of a pro-inflammatory phenotype in the LPS/IFN-γ-treated glial cultures. This effect was observed in the absence of significant cell death but in the presence of impaired metabolic activity.

The toxic effects of MPP+ and rotenone on neurons have repeatedly been demonstrated using primary neuronal cultures, with dopaminergic neurons showing the highest sensitivity to the toxic effects of these compounds. In mouse primary cultures, dopaminergic neuron death is observed at concentrations from 0.1 μM MPP+ (1-week exposure; Kinugawa et al., 2013) or 3 μM MPP+ (48 h exposure; Henze et al., 2005), and 10 nM (1-week exposure; Gao et al., 2003) or 5 nM rotenone (48 h exposure; Radad et al., 2006). Exposure to higher concentrations of these neurotoxins is necessary to induce the death of non-dopaminergic neurons (Gao et al., 2003; Henze et al., 2005). The presence of microglial cells in neuronal cultures has been associated to the increased neurotoxicity of MPP+ and rotenone (Gao et al., 2002, 2003; Emmrich et al., 2013; Kinugawa et al., 2013). However, as MPP+- and rotenone-damaged neurons induce reactive microgliosis, which has a neurotoxic effect, it is difficult to establish the contribution of a direct effect of the toxins on glial cells in the neurotoxicity observed. In fact, although the neurotoxic effect of MPP+ and rotenone has been widely described using *in vivo* and *in vitro* experimental approaches, their direct effects on glial cells remain poorly characterized (Gao et al., 2003; Klintworth et al., 2009; Bournival et al., 2012; Du et al., 2014; Chen et al., 2015; Zhou et al., 2016). Most of the studies performed until now using glial cell cultures have tested whether these neurotoxins induce a pro-inflammatory phenotype in the microglial cells, and the results obtained are controversial. The range of concentrations used in these studies are higher than that used in neuronal cultures (0.1–500 μM MPP+ and 10 nM-1 μM rotenone; Gao et al., 2003; Henze et al., 2005; Klintworth et al., 2009; Bournival et al., 2012; Jin et al., 2012; Du et al., 2014). Some authors have reported no alterations (Klintworth et al., 2009; Ferger et al., 2010; Jin et al., 2012), but others have shown the induction of pro-inflammatory markers in the MPP+- and rotenone-treated microglial cultures (Du et al., 2014; Zhang et al., 2014; Liang et al., 2015; Zhou et al., 2016). Differences in the pattern of neurotoxin treatment (concentration and duration of the treatment) and the cell types used (primary cultures and cell lines from different species) may partially account for the differences observed. Most studies have considered microglial cell lines, while studies using primary microglial cultures are scarce. In this study, we show that concentrations of MPP+ and rotenone that did not affect cell viability in primary glial cultures at 24 h did not result in the induction of a significant pro-inflammatory phenotype (with the exception of IL6 production), but they interfered with the development of the pro-inflammatory phenotype induced by LPS/IFN-γ. Thus, MPP+ and rotenone inhibited pro-inflammatory cytokine production induced by LPS/IFN-γ in glial cells (IL1β in the case of MPP+, and also IL6 and TNFα in the case of rotenone). They also modified the expression of pro-inflammatory enzymes (iNOS, COX2 and/or gp91phox). In general, the alterations observed were more pronounced in the mixed glia than in the microglial cell cultures. In addition, the effect of rotenone was stronger than that of MPP+, although the concentrations of rotenone used were three orders of magnitude below those of MPP+. MPP+ and rotenone treatment also interfered in the phagocytic activity of the microglial cells, which was clearly inhibited after neurotoxin treatment, especially in the presence of LPS/IFN-γ. Altogether, these results suggest that MPP+ and rotenone directly impair the ability of the glial cells to respond to a pro-inflammatory insult. In this sense, exposure to stimuli that affect the mitochondrial activity, such as hypoxia or respiratory chain inhibitors, has been suggested to alter the immune response of macrophages (Wiese et al., 2012).

At the cellular level, the main target of both MPP+ and rotenone is the mitochondrial electron transport chain, where they selectively inhibit complex I (Dauer and Przedborski, 2003). As a consequence, ATP production is compromised, ${\text{O}}_{2}^{-}$ levels increase, and subsequent oxidative stress occurs. This is critical in neuronal cells, where energy production depends mainly on ATP synthesis through oxidative phosphorylation (reviewed in Bélanger et al., 2011). In contrast, astrocytes are mainly glycolytic (reviewed in Bélanger et al., 2011). In addition, astrocytes can generate lactate from glycogen *via* glycolysis under metabolic activation (Hertz et al., 2007). Macrophages/microglia have the capacity to generate ATP by both glycolytic and oxidative pathways (although in the case of microglial cells the field is still underexplored; Van den Bossche et al., 2017; Ghosh et al., 2018). Indeed, they are able to shift from oxidative phosphorylation to aerobic glycolysis (production of lactate in the presence of oxygen) to obtain ATP from different pathways according to the metabolic demands of their activation status (Haschemi et al., 2012; Galván-Peña and O’Neill, 2014; Orihuela et al., 2016). The classical activation or M1/pro-inflammatory phenotype is associated with inhibition of the respiratory chain and the potentiation of aerobic glycolysis, which results in more rapid ATP production to satisfy the metabolic demands associated with the quick pro-inflammatory response of the M1 phenotype. In this situation, the glycolytic and pentose phosphate pathways are potentiated, and oxidative phosphorylation is inhibited (Haschemi et al., 2012). A metabolic-epigenetic crosstalk is suggested to control macrophage activation (Baardman et al., 2015). In contrast, pro-inflammatory stimuli increase tricarboxylic acid activity in astrocytes (Gavillet et al., 2008). In mixed glial cultures, LPS/IFN-γ treatment increased ATP production, an effect that was clearly inhibited by MPP+ and rotenone. These results suggest that MPP+- and rotenone-treated cultures suffer metabolic stress that is aggravated when the cells increase their energetic demands after LPS/IFN-γ treatment. Consequently, activated glial cells may not fulfill their metabolic demands in the presence of MPP+ and rotenone, which would explain why mixed glial cultures exposed to these toxicants were not able to produce an appropriate pro-inflammatory response to LPS/IFN-γ. The effects of MPP+ and rotenone on ATP production were less drastic in the LPS-IFN-γ-treated microglial than the mixed glial cultures. ATP production was not significantly compromised in the LPS/IFN-γ-treated microglial cultures exposed to MPP+ and rotenone, with the exception of 25 μM MPP+ treatment. In addition, the response to LPS/IFN-γ was also impaired, to a lesser extent, in the microglial cultures than in the mixed glial cultures. Altogether, these results suggest that the microglial cells can better cope with the metabolic alterations induced by MPP+ and rotenone than astrocytes can, which accounts for 75% of the cells in the mixed glial cultures. A possible explanation is that while microglial cells developing a pro-inflammatory phenotype switch to glycolysis (Haschemi et al., 2012; Galván-Peña and O’Neill, 2014; Orihuela et al., 2016), astrocytes exposed to pro-inflammatory stimuli increase the activity of the tricarboxylic acid cycle (Gavillet et al., 2008), which in the presence of MPP+ and rotenone will encounter truncated oxidative phosphorylation. However, the involvement of a differential response of the activated microglial cells to the toxins in the presence of astrocytes (or impaired astrocytes) cannot be ruled out.

To assess whether the glycolytic switch mentioned above was behind the ATP production in activated glial cultures, we evaluated the expression of genes encoding critical proteins for the glycolytic pathway. In the microglial cultures, a switch to the glycolytic pathway in the LPS/IFN-γ-activated microglial cultures was suggested by the observed increase in the expression of Glut1, HK1, Pfk1 and Hif1α mRNA and the decreased expression of the Carkl mRNA. Increased expression of the glucose transporter Glut1 may result in more glucose uptake, while the increased expression of Hk1 and Pfk1, which regulate critical steps in glycolysis, may increase the glycolytic rate. It has been suggested that Hif activation contributes to macrophage polarization, and that Hifα-dependent glycolysis favors polarization to a M1 phenotype (Palazon et al., 2016; Taylor et al., 2016). In addition, metabolic intermediates such as succinate play a role in Hif1α stabilization and subsequent IL1β expression in LPS-treated macrophages (Tannahill et al., 2013). Inhibition of the Carkl expression potentiates the flux through the pentose phosphate pathway (Haschemi et al., 2012). Whereas MPP+ exposure resulted in a further increase in Glut1 expression in the LPS/IFN-γ-treated microglial cultures, rotenone exposure partially inhibited the LPS/IFN-γ-induced Pfk and Hifα mRNA expression. Consequently, in the case of microglial cultures, the attenuated pro-inflammatory response to LPS-IFN-γ mostly observed in the presence of rotenone may result from some alterations to the glycolytic switch. In addition, it cannot be ruled out that ATP production through oxidative phosphorylation may also partially contribute to the energy demand required to develop a pro-inflammatory response, even in situations where the switch to glycolysis occurs. In this sense, Wang et al. (2018) showed that 2-deoxyglucose, which blocks glycolysis and partially inhibits glycolytic-dependent oxidative phosphorylation, has a stronger inhibitory effect on the IFN-γ-induced inflammatory response in macrophages than inhibiting glycolysis when replacing glucose in the cell culture medium with galactose, which reduces glycolytic flux without interfering with oxidative phosphorylation. Interestingly, control of the pro-inflammatory macrophage response, through metabolic reprogramming, has been suggested as a potential therapeutic strategy to promote remission in chronic inflammatory diseases (Mills and O’Neill, 2016).

We observed contrasting effects in the LPS/IFN-γ-treated mixed glia and microglial cultures in terms of the mRNA expression of the glycolytic enzymes Glut1, Hk1, and Pfk1. Their expression was inhibited in the mixed glia, suggesting the contribution of astrocytes to the effects observed. Although astrocytes are mainly glycolytic (Bélanger et al., 2011), pro-inflammatory stimuli increase tricarboxylic acid activity in astrocytes (Gavillet et al., 2008). The decreased expression of the glycolytic enzymes we observed may reflect this switch. Consequently, the impaired response of the mixed glial cultures to LPS/IFN-γ in the presence of MPP+ and rotenone may be explained by the fact that oxidative phosphorylation, which would be responsible for the main ATP production from products of the tricarboxylic acid cycle, is inhibited by MPP+ and rotenone. However, the involvement of the microglial cells in the response of the mixed glial cultures to MPP+ and rotenone plus LPS/IFN-γ cannot be ruled out.

Finally, some studies show that MPP+ causes DNA damage (Zhang et al., 1995) and oxidative DNA damage in neuronal cells (Chen et al., 2005). In addition, rotenone-induced DNA damage (Goswami et al., 2016) and DNA methylation (Scola et al., 2014) in neurons have also been described. Although there are no reports on MPP+- and rotenone-induced DNA alterations on glial cells, we cannot discard that these alterations may be behind the decreased expression of inflammatory markers we detected in LPS/IFN-γ–treated glial cultures exposed to the neurotoxins.

## Conclusion

In summary, the results of the present study show that the pro-inflammatory response induced by LPS/IFN-γ in mouse primary glial cell cultures, is impaired under MPP+ and rotenone exposure, mainly when both the astrocytes and microglia are present. This suggests that the immune response of the glial cells is compromised in the presence of neurotoxins that inhibit the mitochondrial electron transport chain. We are currently studying the possible effects of MPP+ and rotenone on the development of an anti-inflammatory phenotype by the glial cells. Although the involvement of the glial cells in the development of neurodegenerative diseases is widely accepted, the precise role they play in every neurodegenerative disorder remains to be established. As many genetic and environmental factors are probably involved in the etiopathogenesis of neurodegenerative diseases, many factors may also determine when and how the glial cells take part in the pathological process. In the case of pathologies where the exposure to certain neurotoxicants is a risk factor, such as PD, the direct effect of the toxic agents on glial cell function may be an additional factor to take into account, as alterations in glial function will have an effect on neuronal function and CNS homeostasis. In this context, our results suggest that glial metabolic alterations induced by neurotoxin exposure compromises the brain’s immune response. This impaired immune response may imply a more vulnerable brain, which can be a further aspect contributing to the development of PD.

## Data Availability

All data generated or analyzed during this study are included in this published article.

## Author Contributions

NR-L and EX-E performed most of the experiments and analyzed the data. JSe participated in the processing of the samples and quantified the phagocytosis assay. JSa provided critical guidance and contributed to the final version of the manuscript. CS conceived and coordinated the experiments, provided guidance in the production of data and drafted the manuscript. All authors provided input and ideas throughout the process, and critically revised and approved the final manuscript.

## Conflict of Interest Statement

The authors declare that the research was conducted in the absence of any commercial or financial relationships that could be construed as a potential conflict of interest.

## Abbreviations

ATP: adenosine triphosphate
BSA: bovine serum albumin
(COX-2): cyclooxygenase-2
Carkl: carbohydrate kinase-like protein
CNS: central nervous system
Glut: glucose transporter
gp91phox: NADPH oxidase, catalytic subunit
Hif: hypoxia-inducible factor
Hk: hexokinase
IL: interleukin
IFN-γ: interferon-γ
iNOS: inducible nitric oxide synthase
LPS: lipopolysaccharide
MPP+: 1-methyl-4-phenylpyridinium
MPTP: 1-methyl-4-phenyl-1,2,3,6-tetrahydropyridine
MTT: 3-(4,5-dimethylthiazol-2-yl)-2,5-diphenyltetrazolium bromide
NO: nitric oxide
PBS: Phosphate-buffered saline
PCR: polymerase chain reaction
PFK-P: phosphofructokinase
PI: propidium iodide
PD: Parkinson’s disease
qRT-PCR: quantitative real time PCR
Rot: rotenone
TNF: tumor necrosis factor.
